# Safety and efficacy of hydroxyurea and eflornithine against most blood parasites *Babesia* and *Theileria*

**DOI:** 10.1371/journal.pone.0228996

**Published:** 2020-02-13

**Authors:** Gaber El-Saber Batiha, Amany Magdy Beshbishy, Oluyomi Stephen Adeyemi, Eman Nadwa, Eman Rashwan, Naoaki Yokoyama, Ikuo Igarashi

**Affiliations:** 1 National Research Center for Protozoan Diseases, Obihiro University of Agriculture and Veterinary Medicine, Obihiro, Hokkaido, Japan; 2 Department of Pharmacology and Therapeutics, Faculty of Veterinary Medicine, Damanhour University, Damanhour, Al-Beheira, Egypt; 3 Department of Biochemistry, Medicinal Biochemistry, Nanomedicine and Toxicology Laboratory, Landmark University, Omu-Aran, Kwara State, Nigeria; 4 Department of Pharmacology and Therapeutics, College of Medicine, Jouf University, Jouf, Egypt; 5 Department of Medical Pharmacology, Faculty of Medicine, Cairo University, Cario, Egypt; 6 Department of Physiology, College of Medicine, Al-Azhar University, Assuit, Egypt; 7 Department of Physiology, College of Medicine, Jouf University, Sakaka, Saudi Arabia; University of Agricultural Sciences and Veterinary Medicine Cluj-Napoca, Life Science Institute, ROMANIA

## Abstract

**Background:**

The plenteous resistance to and undesirable consequences of the existing antipiroplasmic therapies have emphasized the urgent need for new chemotherapeutics and drug targets for both prophylaxis and chemotherapy. Hydroxyurea (HYD) is an antineoplastic agent with antitrypanosomal activity. Eflornithine (α-difluoro-methyl ornithine, DFMO) is the best choice therapy for the treatment of late-stage Gambian human African trypanosomiasis.

**Methods:**

In this study, the inhibitory and combination efficacy of HYD and DFMO with existing babesicidal drugs (diminazene aceturate (DA), atovaquone (ATV), and clofazimine (CLF)) deoxyribonucleotide *in vitro* against the multiplication of *Babesia* and *Theileria*. As well as, their chemotherapeutic effects were assessed on *B*. *microti* strain that infects rodents. The Cell Counting Kits-8 (CCK-8) test was used to examine their cytotoxicity on human foreskin fibroblast (HFF), mouse embryonic fibroblast (NIH/3T3), and Madin–Darby bovine kidney (MDBK) cells.

**Findings:**

HYD and DFMO suppressed the multiplication of all tested species (*B*. *bigemina*, *B*. *bovis*, *B*. *caballi*, *B*. *divergens*, and *T*. *equi*) in a dose-related manner. HFF, NIH/3T3, or MDBK cell viability was not influenced by DFMO at 1000 μM, while HYD affected the MDBK cell viability at EC_50_ value of 887.5±14.4 μM. The *in vitro* combination treatments of DFMO and HYD with CLF, DA, and ATV exhibited synergistic and additive efficacy toward all tested species. The *in vivo* experiment revealed that HYD and DFMO oral administration at 100 and 50 mg/kg inhibited *B*. *microti* multiplication in mice by 60.1% and 78.2%, respectively. HYD-DA and DFMO-DA combined treatments showed higher chemotherapeutic efficacy than their monotherapies.

**Conclusion:**

These results indicate the prospects of HYD and DFMO as drug candidates for piroplasmosis treatment, when combined mainly with DA, ATV, and CLF. Therefore, further studies are needed to combine HYD or DFMO with either ATV or CLF and examine their impact on *B*. *microti* infection in mice.

## Introduction

Numerous drugs have been used for several years for piroplasmosis treatment, including diminazene aceturate (DA), atovaquone (ATV), oxytetracycline, and azithromycin, and have increasingly proven to be ineffective because of their toxicity and advanced resistance [1, 2]. In the wake of resistance and toxicity to the available drug options, new drug candidates *inter alia* epoxomicin, allicin, nerolidol, triclosan, gossypol, nitidine chloride, 17-DMAG, *trans*-chalcone, ellagic acid, and ivermectin have been evaluated but none has yet been passed for clinical trials [3–7]. Further yet, Batiha et al. [8], as well as Beshbishy et al. [9], documented the babesicidal effects of herbal extracts that showed no side effects to the host. Overall, it is clear that the development of treatment options for piroplasmosis is vital for improving disease treatment and control. Regrettably, the process for the development and approval of a new drug is tedious. Alternatively, screening drugs on the market for repurposing might hasten the process to provide treatment choices for bovine, equine piroplasmosis as well as human babesiosis. The extensive socio-economic and welfare effects of bovine and equine piroplasmosis on animals and human babesiosis on humans have sustained the demand for pharmaceutical advancements to develop novel drug candidates [2].

Hydroxyurea (HYD), is an antimetabolite and antineoplastic agent that is used alone or in combination with itraconazole, clotrimazole, and terbinafine for the treatment of several kinds of cancer and fungal infections [10, 11]. It increases fetal hemoglobin concentration and thus decreases the prevalence of severe health crises and urgent blood transmissions in sickle cell anemia patients. HYD, a selective inhibitor of ribonucleotide reductase [12], the main enzyme responsible for ribonucleotide, diphosphates conversion to deoxy ribonucleotide diphosphates, thus inhibiting cells outgoing from the G1/S stage of the cell cycle [13, 14]. Cokic et al. [15] documented that HYD is well absorbed *in vivo*, converted to free radical nitroxide and transferred to the cells, whereas the tyrosyl free radical suppression happens at ribonucleotide reductase active site, thereby inhibiting the enzyme. Moreover, HYD has been documented as the best choice for anemic disease treatment such as β-thalassemia [16]. HYD reportedly possesses an antiparasitic activity through blocking *Toxoplasma gondii* tachyzoite replication [17] and synchronizing the kinetoplast DNA of several parasites, such as *Crithidia fasciculate*, *Leishmania major*, *L*. *infantum*, *L*. *tarentolae*, *Trypanosoma brucei*, and *T*. *cruzi* [16, 18].

Eflornithine (α-difluoro-methyl ornithine, DFMO) is a fluoroamino analogue of ornithine, an amino acid present in all living species and used in the urea cycle to eliminate excess nitrogen from the body [19]. DFMO is the marketable drug for the treatment of late-stage Gambian human African trypanosomiasis (sleeping sickness) and it is manufactured by Sanofi Aventis and sold in the USA under the brand name Ornidyl^®^ [20]. It has shown a remarkable antitrypanosomal efficacy, low systemic toxicity [21], and high tolerability—even when given to children in relatively high dosages—compared with melarsoprol, the standard treatment for trypanosomiasis [22]. DFMO inhibits ornithine decarboxylase, the primary enzyme required for polyamines putrescine and spermidine synthesis, which is needed for cell multiplication and differentiation and it enters trypanosomes through the amino acid transporter AAT6 [23]. Notably, DFMO also has anabolic, wound-healing, and immuno-enhancing effects as well as improves liver function and helps in the detoxification of harmful substances [24, 25].

Although HYD and DFMO have been studied for their antiparasitic activity against several protozoan parasites, there have no reports on their antipiroplasmic efficacy. It is against this backdrop that the current study aimed to investigate the growth-inhibitory efficacy of HYD and DFMO as well as their combined effect with DA, ATV, and clofazimine (CLF) on *B*. *bovis*, *B*. *bigemina*, *B*. *divergens*, *B*. *caballi* and *T*. *equi* multiplication *in vitro*. In addition to the investigation of their chemotherapy prospects against *B*. *microti*-infected mice.

## Materials and methods

### Chemical reagents

Stock solutions (10 mM) in dimethyl sulfoxide (DMSO) of hydroxyurea (HYD; CH_4_N_2_O_2_), DL-α-difluoro-methyl ornithine hydrochloride hydrate (DFMO; C_6_H_12_F_2_N_2_O_2_ · xHCl · yH_2_O), DA, CLF, and ATV (Sigma-Aldrich, Japan) were stored at –30°C and used for babesicidal evaluation. Reference drugs including DA, CLF, and ATV were used either individually or combined with HYD or DFMO for both the *in vivo* and *in vitro* experiments. For the fluorescence assay, SYBR Green I (SGI) stain (10,000×, Lonza, USA) was mixed with the lysis buffer containing saponin (0.016% w/v), EDTA (10 mM), Triton X–100 (1.6% v/v), and Tris (130 mM at pH 7.5) which was filtered using a polyethersulfone (0.22 μm) and kept at 4°C.

### Cultivation condition *in vitro*

#### Parasites and mice

*Babesia* parasites were incubated and maintained at 37°C in a humidified chamber under 5% CO_2_, 5% O_2_, and 90% N_2_ atmosphere using a microaerophilic stationary-phase culture for conducting the *in vitro* experiment [26]. Briefly, *Babesia divergens* Germany strain was cultured in cattle red blood cells (RBCs, collected from cattle farm of Obihiro University of Agriculture and Veterinary Medicine and stored at 4°C) in Roswell Park Memorial Institute 1640 (RPMI 1640; Sigma-Aldrich, Tokyo, Japan) medium replenished with 40% cattle serum, while culture medium 199 (M199; Sigma-Aldrich, Tokyo, Japan) was used for the *B*. *bigemina* Argentina strain and *B*. *bovis* Texas strain, and *T*. *equi* USDA strain cultured in cattle RBCs supplemented with 40% cattle serum and horse RBCs (collected from horse farm of Obihiro University of Agriculture and Veterinary Medicine and stored at 4°C) maintained in hypoxanthine (MP Biomedicals, USA; final concentration 13.6 μg/mL) and 40% horse serum, respectively [7]. GIT medium supplemented with 40% horse serum was used as a growth medium for *B*. *caballi* USDA strain cultured in horse RBCs. To ensure free-bacterial contamination, amphotericin B (0.15 μg/mL) (Sigma-Aldrich, USA), streptomycin (60 U/mL), and penicillin G (60 U/mL) were added to all media.

For the *in vivo* study, two female BALB/c mice obtained from CLEA Japan were preliminarily given an intraperitoneal injection of Munich strain *B*. *microti* (retrieved from the stock stored at –80°C), and the observation of parasitemia was performed as previously described elsewhere [8, 27].

#### Ethical approval

The experiments described in this study were performed, and RBCs were collected from cattle and equine farm of Obihiro University of Agriculture and Veterinary Medicine in accordance with the local guidelines for animal experimentation, as approved by the Obihiro University of Agriculture and Veterinary Medicine, Japan (accession numbers 28-111-2, 28–110, and 1417–2). This ethical approval was developed through the basic guidelines for the proper conduct of animal experimentation and related activities in Academic Research Institutions, Ministry of Education, Culture, Sports and Technology (MEXT), Japan.

#### The inhibition assay of HYD and DFMO *in vitro*

The *Babesia* fluorescent assay was carried out on the *in vitro* culture, as previously reported elsewhere [3, 4]. Briefly, in three separate trials, using two-fold dilutions, different concentrations of HYD, DFMO, DA, CLF, and ATV were prepared in the culture medium and added in 96-well plates in triplicate with 1% parasitemia for *T*. *equi*, *B*. *caballi* and *B*. *divergens* at 5% hematocrit (HCT) while for *B*. *bigemina* and *B*. *bovis* using 2.5% HCT. Afterward, parasite cultures were incubated for 4 consecutive days without changing medium at 37°C humidified multi-gas incubator in 5% CO_2_, 5% O_2_, and 90% N_2_ atmosphere. On day four of culture, an aliquot (100 μL) of lysis buffer mixed with 0.2 μl/ml SG1 was added per well and the fluorescence readings were acquired on a spectrofluorimeter (Fluoroskan Ascent, Thermo Fisher Scientific, USA) with an excitation wavelength of 485 nm and an emission wavelength of 518 nm.

#### Parasite viability test *in vitro*

The viability studies of HYD- and DFMO-treated parasite were monitored via microscopy, as reported elsewhere [3]. In a 96-well microtiter plate, a reaction volume of 200 μL containing 180 μL of each specific media containing various concentrations of HYD, DFMO, and DA and 20 μL of 1% parasitemia of iRBCs were incubated at 37°C for 4 days in a humidified incubator. The parasitemia was monitored in Giemsa-stained thin blood smears in 2000 RBCs every 24h, 48h, 72h, and 96h. On the fifth day, a mixture of iRBCs (3 μL) from each well and fresh equine or bovine RBCs (7 μL) was transferred to another plate cultured in a medium free from drug and then left for an additional 6 days and parasitemia monitoring occurred via microscopy as reported elsewhere [3].

#### *In vitro* efficacy of the drug combination treatment

In parallel with the single-treatment assay, the combined efficacy of HYD and DFMO with DA, CLF, and ATV was examined using the fluorescence inhibition assay, as reported previously elsewhere [28]. Five selected concentrations (0.25 ×, 0.5 ×, 1 ×, 2 × and 4 × the IC_50_) of HYD or DFMO with DA or ATV or CLF (S1 Table) were set up in three sets of duplicate wells. The drug cultivation and the fluorescence values were detected as described above.

#### Evaluation of the impacts of HYD and DFMO on RBCs of cattle and horse

Prior to parasite subculture, various concentrations (10, 200, and 600 μM) of HYD and DFMO were mixed with fresh bovine and equine RBCs and incubated at a humidified incubator for 3 h. Afterward, the pretreated-RBCs were mixed with *B*. *bovis* and *T*. *equi*-infected RBCs (iRBCs) after washing thrice with PBS to achieve 1% parasitemia. Thereafter, using a 24-well plate, an aliquot of iRBCs (100 μL) was mixed with culture media (900 μL) the parasitemia was monitored as described above.

### Cytotoxicity assay

#### Cultures of normal cell lines

Cultures of Human foreskin fibroblast (HFF; HFF-1 ATCC^®^ SCRC-1041^™^), Madin–Darby bovine kidney (MDBK; ECACC) and mouse embryonic fibroblast (NIH/3T3; ATCC^®^ CRL-1658^™^) cells were retrieved from -80°C stock and cultured continuously at 37°C under atmosphere 5% CO_2_ in our laboratory. The NIH/3T3 and HFF cell lines were maintained in Dulbecco Modified Eagle’s Medium (DMEM; Gibco, Grand Island, NY, USA), while MDBK cell line grown in Minimum Essential Medium Eagle (MEM; Gibco) and cell cultivation was performed as describe everywhere [3, 4].

#### Cytotoxic action of HYD, DFMO, DA, CLF, and ATV on normal cells

The cell viability test was conducted in a 96-well plate as described elsewhere [3, 4]. Briefly, an aliquot of (100 μL) cells was implanted at a concentration of 5×10^4^ cells/mL in DMEM or MEM with fetal bovine serum and incubated overnight under atmosphere 5% CO_2_ at 37°C for attachment. Using two-fold dilutions, aliquots (10 μL) of drugs were added in triplicate to each well to attain final concentrations of 50 to 1000 μM and incubated for an additional 24 h. Thereafter, Cell Counting Kits-8 (CCK-8) (10 μL) was added, and the absorbance was measured at 450 nm [4].

### *In vivo* experiments

#### *In vivo* chemotherapeutic effects of HYD and DFMO

HYD and DFMO were examined for their *in vivo* chemotherapeutic efficacy using *B*. *microti*–infected BALB/c mice according to a procedure described elsewhere [8, 9]. Briefly, 25 female eight-week-old mice were placed in an environment free from pathogens with 22°C temperature and adjusted humidity and under 12 h light and 12 h darkness and randomly distributed into five groups. The mice in groups 2 through 5 obtained 500 μL of 1×10^7^ *B*. *microti* iRBC by intraperitoneal (i.p.) injection. Group 1 served as a negative control and was neither infected nor treated. At 1% parasitemia, drug treatment of the mice by i.p. started, continuing for 5 days. Group 2 act as a positive control group and received 95% DDW and 5% DMSO. Group 3 served as a reference to drug control and received 25 mg/kg body weight (BW) of DA. Groups 4 and 5 received HYD (50 mg/kg BW) and DFMO (25 mg/kg BW), respectively.

Thirty-five mice, randomly distributed into seven groups, received inoculum of 1 × 10^7^ *B*. *microti*-iRBCs by i.p injection were used to verify the *in vivo* efficacy of oral administration and the combinations of HYD and DFMO with DA. Groups 1 and 2 represent the negative and positive control ones, respectively. Groups 3 to 5 received i.p. injection of DA (25 mg/kg BW), 100 mg/kg BW of HYD, and 50 mg/kg BW DFMO by an oral route, respectively, while the sixth and seventh groups received combinations of 12.5 mg/kg BW DA + 50 mg/kg BW HYD and 12.5 mg/kg BW DA + 25 mg/kg BW DFMO, respectively, by intraperitoneal and oral routes continuing for 5 days. The parasitemia was monitored by preparing Giemsa-stained smears every 2 days in about 5000 RBCs by microscopy until day 53. Furthermore, the hematological parameters, including hemoglobin (HGB), RBCs, and hematocrit (HCT), were determined every 4 days using an automatic hematology analyzer (Celltac α MEK-6450, Nihon Kohden, Japan). At the end of the *in vivo* experiment, an anesthetic system using an inhaler containing isoflurane was used to euthanize all mice by placing them in the induction chamber, adjusting the oxygen flowmeter to 0.8 to 1.5 L/min and vaporizer to 3% to 5%. When mice were completely anesthetized, all of them were killed by cervical dislocation according to the ethical approval confirmed by the Basic Guidelines for Proper Conduct of Animal Experiment and Related Activities in Academic Research Institutions, the Ministry of Education, Culture, Sports and Technology (MEXT), Japan.

### Statistical analysis

The IC_50_ values of HYD, DFMO, ATV, CLF, and DA were determined from the *in vitro* growth inhibition by nonlinear regression curve fit on a GraphPad Prism (GraphPad Software Inc., USA). CompuSyn software was used for combination index (CI) values calculation, and the synergetic degree was established as the average weighted CI values by using the following formulae; ((1 × IC_50_) + (2 × IC_75_) + (3 × IC_90_) + (4 × IC_95_))/10 and the resulted values were demonstrated using the recommended CI scale developed previously (S2 Table) [28]. The significant variations (*P* < 0.05) among group mean values on parasitemia and one-way ANOVA Tukey’s test in GraphPad Prism version 5.0 was used to analyze hematology profiles in mice infected with *B*. *microti*.

## Results

### Growth -inhibition efficacy of HYD and DFMO *in vitro*

**Fig 1:**
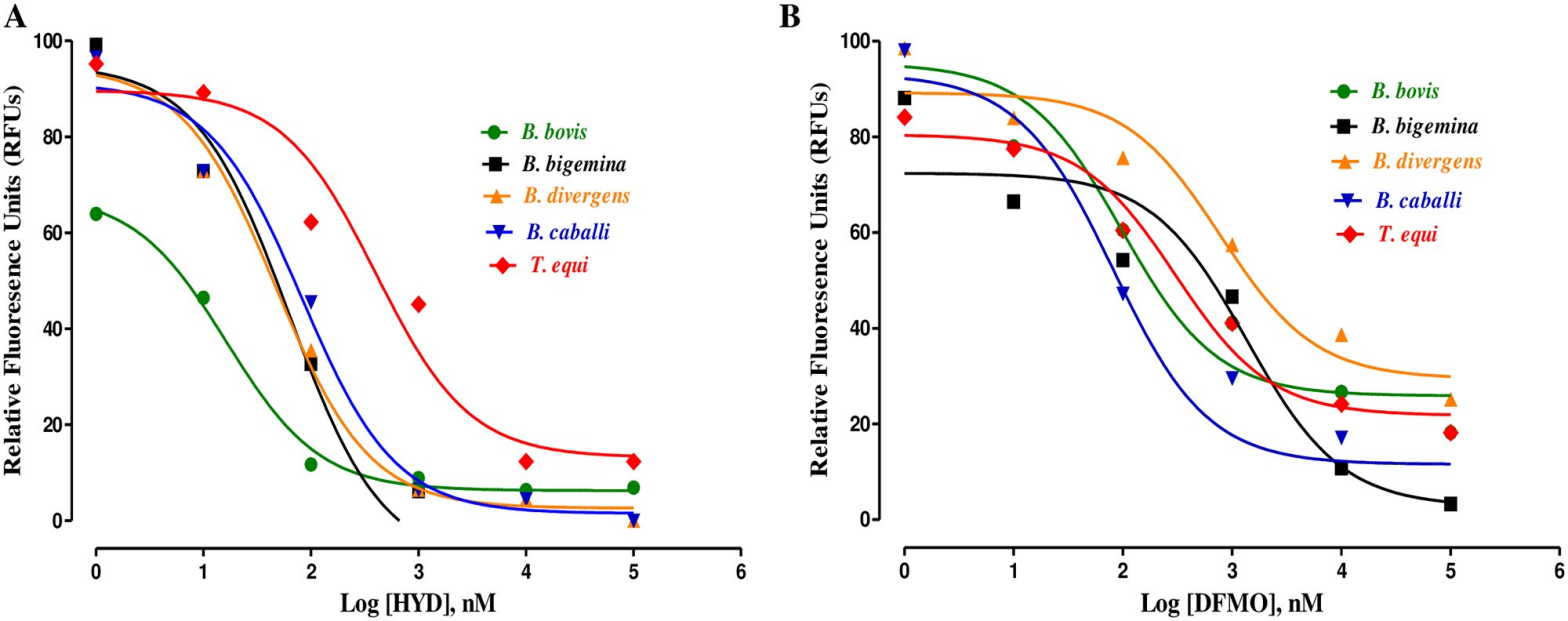
The relationship between the relative fluorescence units (RFUs) and the log concentrations of HYD (nM) (A) and DFMO (nM) (B) on *B*. *bovis*, *B*. *bigemina*, *B*. *divergens*, *B*. *caballi* and *T*. *equi*. The non-linear regression (curve fit analysis) in GraphPad Prism software (GraphPad Software Inc. USA) was used to calculate the IC_50_’s. The percentage of parasite growth inhibitory efficacy is calculated as the percentage of parasites inhibited divided by that of the positive control wells, and the result was subtracted from the negative control wells.

The *in vitro*-inhibition test revealed that HYD and DFMO significantly restricted (*P* < 0.05) the multiplication of *B*. *bovis*, *B*. *bigemina*, *B*. *divergens*, *B*. *caballi*, and *T*. *equi* in a dose-related manner. *B*. *bovis* multiplication was suppressed significantly (t-test: *t*_(5)_ = 10.30, *P* = 0.0001) at 3.125 μM HYD, whereas *T*. *equi*, *B*. *divergens*, *B*. *bigemina* and *B*. *caballi* multiplication was suppressed significantly (t-test: *t*_(5)_ = 4.428, *P* = 0.005) at 6.25 μM HYD (Fig 1A). DFMO inhibited the *in vitro* multiplication of *B*. *bovis*, *B*. *divergens* (t-test: *t*_(5)_ = 3.859, *P* = 0.01), *B*. *bigemina* and *T*. *equi* significantly (t-test: *t*_(5)_ = 5.329, *P* = 0.005) at 6.25 μM. DFMO inhibited the multiplication of *B*. *caballi* significantly (t-test: *t*_(5)_ = 7.456, *P* < 0.0001) at 6.25 μM (Fig 1B).

HYD and DFMO suppressed *B*. *bovis*, *B*. *bigemina*, *B*. *divergens*, *B*. *caballi*, and *T*. *equi* multiplication at IC_50_ values shown in Table 1.

**Table 1:** IC_50_ and selective index values of HYD and DFMO.

Drug	Parasite	IC_50_ (μM)^a^	EC_50_ (μM)^b^			Selective indices^c^		
			MDBK	NIH/3T3	HFF	MDBK	NIH/3T3	HFF
**HYD**	*B*. *bovis*	**85.2 ± 9.4**	**887.5± 14.4**	**˃ 1000**	**˃1000**	**10.4**	**˃ 11.7**	**˃ 11.7**
	*B*. *bigemina*	**68.7 ± 6.3**	**887.5± 14.4**	**˃ 1000**	**˃ 1000**	**12.9**	**˃ 14.6**	**˃ 14.6**
	*B*. *divergens*	**57.3 ± 4.3**	**887.5± 14.4**	**˃ 1000**	**˃ 1000**	**15.5**	**˃ 17.5**	**˃ 17.5**
	*B*. *caballi*	**49.5 ± 2.3**	**887.5± 14.4**	**˃ 1000**	**˃ 1000**	**17.9**	**˃ 20.2**	**˃ 20.2**
	*T*. *equi*	**19.6 ± 1.4**	**887.5± 14.4**	**˃ 1000**	**˃ 1000**	**45.3**	**˃ 51**	**˃ 51**
**DFMO**	*B*. *bovis*	**98 ± 3.3**	**˃ 1000**	**˃ 1000**	**˃1000**	**˃ 10.2**	**˃ 10.2**	**˃ 10.2**
	*B*. *bigemina*	**79 ± 4.5**	**˃ 1000**	**˃ 1000**	**˃ 1000**	**˃ 12.7**	**˃ 12.7**	**˃ 12.7**
	*B*. *divergens*	**46.9 ± 1.4**	**˃ 1000**	**˃ 1000**	**˃ 1000**	**˃ 21.3**	**˃ 21.3**	**˃ 21.3**
	*B*. *caballi*	**71 ± 2.6**	**˃ 1000**	**˃ 1000**	**˃ 1000**	**˃ 14.1**	**˃ 14.1**	**˃ 14.1**
	*T*. *equi*	**97 ± 5.6**	**˃ 1000**	**˃ 1000**	**˃ 1000**	**˃ 10.3**	**˃ 10.3**	**˃ 10.3**

^a^ IC_50_ values of HYD and DFMO on all tested parasites *in vitro*.

^b^ EC_50_ values of HYD and DFMO on the tested cell lines. The dose-response curve using nonlinear regression (curve fit analysis) was used to detect all of these values. The values obtained from the means of triplicate experiments.

^c^ Selective index calculated as the ratio of the EC_50_ of cell lines to the IC_50_ of each parasite.

In the present study, DA, ATV, and CLF restricted *B*. *bovis*, *B*. *bigemina*, *B*. *divergens*, *B*. *caballi*, and *T*. *equi* multiplication at IC_50_ values shown in S3 Table. The diluent used did not affect the efficacy of HYD and DFMO was not influenced as no significant variation in inhibition between the positive and negative wells. The preliminary evaluation of HYD and DFMO was performed to detect their efficacy on host RBCs prior to *B*. *bovis* and *T*. *equi* subculture, bovine and equine RBCs were incubated for 3 h with HYD and DFMO to a final concentration of 600 μM. The parasite proliferation did not significantly differ between the *B*. *bovis* (S1 Fig) or *T*. *equi* RBCs (S2 Fig) treated with either HYD or DFMO and the untreated one for either species.

### Parasite viability after treatment with HYD or DFMO

A viability assay revealed that HYD at a concentration of 2×IC_50_ completely suppressed *B*. *caballi* and *T*. *equi* multiplication, whereas 4×IC_50_ concentration cleared *B*. *divergens*, *B*. *bigemina*, and *B*. *bovis*. All tested DFMO-treated parasites completely suppressed at 4×IC_50_ except *B*. *divergens* cleared at 2×IC_50_ concentration (Table 2).

**Table 2:** Viability of parasites treated with HYD and DFMO.

**Drug**	**Conc. of compound**	**Parasites**				
		***B*. *bovis***	***B*. *bigemina***	***B*. *divergens***	***B*. *caballi***	***T*. *equi***
**HYD**	**0.25×IC**_**50**_	**+**	**+**	**+**	**+**	**+**
	**0.5×IC**_**50**_	**+**	**+**	**+**	**+**	**+**
	**1×IC**_**50**_	**+**	**+**	**+**	**+**	**+**
	**2 ×IC**_**50**_	**+**	**+**	**+**	**-**	**-**
	**4 ×IC**_**50**_	**-**	**-**	**-**	**-**	**-**
		***B*. *bovis***	***B*. *bigemina***	***B*. *divergens***	***B*. *caballi***	***T*. *equi***
**DFMO**	**0.25×IC**_**50**_	**+**	**+**	**+**	**+**	**+**
	**0.5×IC**_**50**_	**+**	**+**	**+**	**+**	**+**
	**1×IC**_**50**_	**+**	**+**	**+**	**+**	**+**
	**2 ×IC**_**50**_	**+**	**+**	**-**	**+**	**+**
	**4 ×IC**_**50**_	**-**	**-**	**-**	**-**	**-**
	**Negative control**	**+**	**+**	**+**	**+**	**+**

Results are calculated as the mean values from three separate trials ± SD, a positive (+) indicates parasites regrowth, and a negative (-) shows the parasites total clearance after drug pressure withdrawal using microscopy assay.

HYD, hydroxyurea; DFMO, eflornithine.

### *In vitro* potential of the combination of HYD or DFMO with DA, ATV, or CLF

The HYD–DA combined treatment was additive toward *B*. *bovis* and synergistic toward the other four species. The HYD–ATV combined treatment was synergistic against all tested parasites except *B*. *bovis*, which showed an additive effect. The HYD–CLF combined treatment was synergistic toward *B*. *bovis*, *B*. *bigemina*, *B*. *divergens*, and *T*. *equi*, but additive toward *B*. *caballi*. The DFMO–DA combined treatment revealed synergistic efficacy toward *T*. *equi*, *B*. *caballi*, and *B*. *bigemina*, but additive toward *B*. *divergens* and *B*. *bovis*. The DFMO–ATV combined treatment revealed synergistic efficacy toward all tested species. The DFMO–CLF combined treatment was synergistic toward *T*. *equi*, *B*. *divergens*, and *B*. *bovis*, but additive toward *B*. *caballi* and *B*. *bigemina* (Table 3).

**Table 3:** Combination effect of HYD or DFMO with either DA, ATV, or CLF *in vitro*.

Drug combination		Parasites				
		*B*. *bovis*	*B*. *bigemina*	*B*. *divergens*	*B*. *caballi*	*T*. *equi*
**HYD + DA**	**CI value**	1.1014	0.8336	0.8502	0.7512	0.7710
	**Interaction**	**Additive**	**Synergistic**	**Synergistic**	**Synergistic**	**Synergistic**
**DFMO + DA**	**CI value**	1.101	0.6712	1.0223	0.8520	0.7022
	**Interaction**	**Additive**	**Synergistic**	**Additive**	**Synergistic**	**Synergistic**
**HYD + ATV**	**CI value**	1.0455	0.3470	0.7046	0.8164	0.6750
	**Interaction**	**Additive**	**Synergistic**	**Synergistic**	**Synergistic**	**Synergistic**
**DFMO + ATV**	**CI value**	0.3873	0.5180	0.8730	0.7348	0.6589
	**Interaction**	**Synergistic**	**Synergistic**	**Synergistic**	**Synergistic**	**Synergistic**
**HYD + CLF**	**CI value**	0.6171	0.5692	0.4342	0.9126	0.8150
	**Interaction**	**Synergistic**	**Synergistic**	**Synergistic**	**Additive**	**Synergistic**
**DFMO + CLF**	**CI value**	0.2771	1.104	0.6158	1.0238	0.5470
	**Interaction**	**Synergistic**	**Additive**	**Synergistic**	**Additive**	**Synergistic**

HYD, hydroxyurea; DFMO, eflornithine; DA, diminazene aceturate; ATV, atovaquone; CLF, clofazimine; CI, combination index

### Toxicity of HYD, DFMO, DA, ATV, and CLF on normal cells

The cytotoxicity assay of HYD and DFMO was assessed on HFF, NIH/3T3, and MDBK cell lines (Table 1). DFMO did not reduce HFF, NIH/3T3, or MDBK cell viability at a concentration of 1000 μM, whereas HYD at 1000 μM suppressed the viability of MDBK cell line at EC_50_ of 887.5 ± 14.4 μM (Table 1). Regarding the reference babesicidal drugs, neither DA nor ATV (final concentration 100 μM) inhibited the viability of HFF, NIH/3T3, or MDBK cell lines, while CLF in EC_50_ of 34 ± 3.4 μM reduced the MDBK cell viability. The highest selective index values (ratio of the EC_50_ on the cell cultures to the IC_50_ on the parasites) for HYD were 45.3, ˃ 51, and ˃ 51 times toward *T*. *equi* versus MDBK, NIH/3T3, and HFF cells, respectively. For DFMO, the highest selectivity index was ˃ 21.3 times toward *B*. *divergens* versus the HFF, NIH/3T3, and MDBK cells (Table 1).

### The *in vivo* chemotherapeutic potential of HYD and DFMO in mice

**Fig 2:**
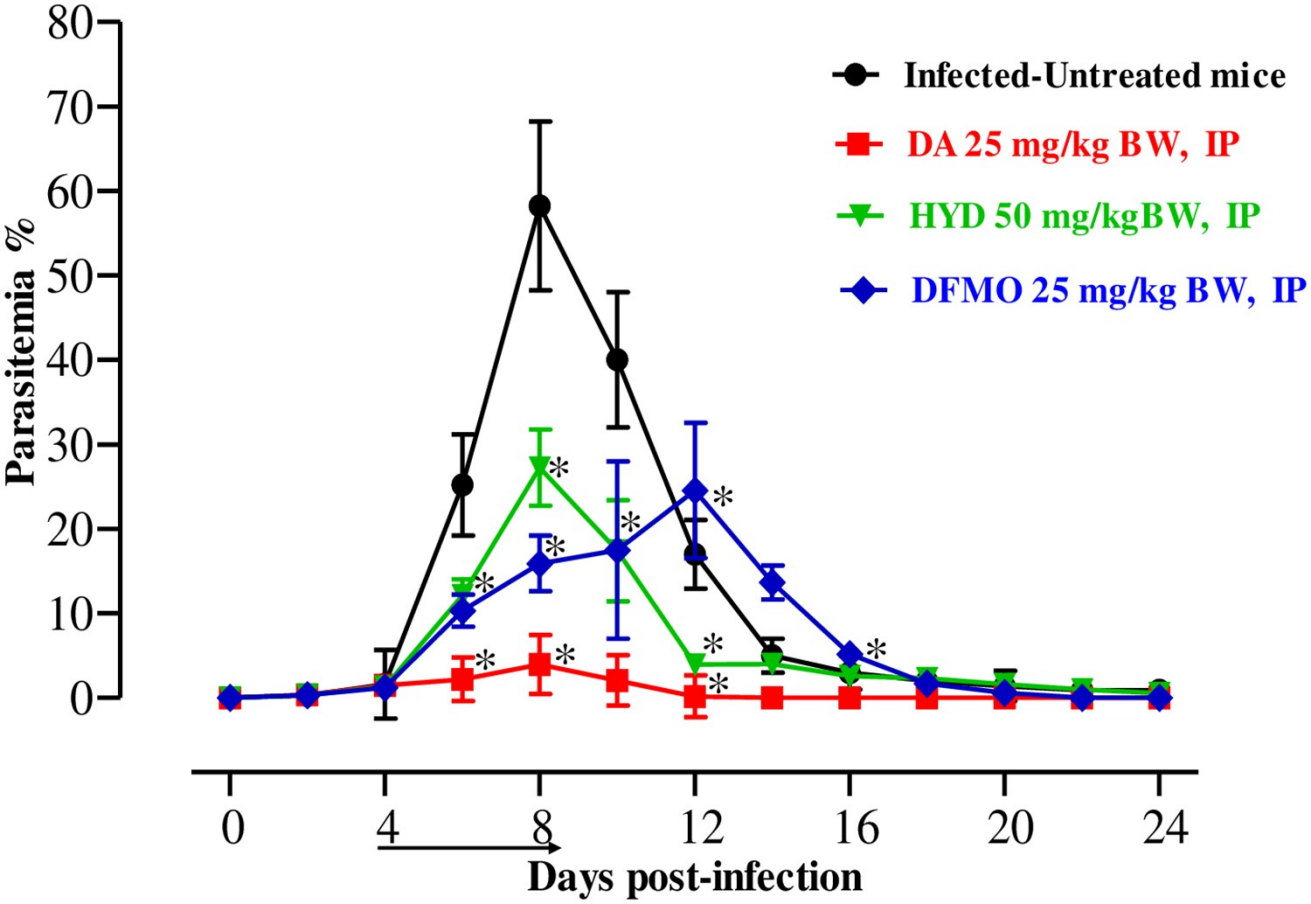
*In vivo* chemotherapeutic potential of HYD and DFMO on *B*. *microti*. Graph reveals the chemotherapeutic potential of DA-IP, HYD-IP, and DFMO-IP compared to the infected-untreated group. The arrow shows 5 successive days of drug administration starting from day 4 to 8 p.i. The asterisks (*) show the significant variation (*P* < 0.05) between drug-treated and positive groups. Parasitemia was detected using Giemsa-stained thin blood smears by counting iRBCs among 5000 RBCs.

To examine the *in vivo* chemotherapeutic potential of HYD and DFMO, female BALB/c mice were affected by *B*. *microti*, and the two drugs were administered for 5 days after the infection reach 1% parasitemia. On the eighth day post-infection (p.i.), the DDW control group showed rapid parasitemia growth reached 58.2% (Fig 2) and the parasitemia reduced slowly on the subsequent days.

The level of parasitemia in all treated groups was reduced at a statistically significant lower percentage of parasitemia than that of the control group (ANOVA: *F*_(1.448, 6.236)_ = 5.784, *P* = 0.001 for 50 mg/kg HYD; ANOVA: *F*_(1.159, 4.969)_ = 5.784, *P* = 0.001 for 25 mg/kg DFMO) from days 6 to 12 p.i. The level of peak parasitemia in 25 mg/kg DA, 25 mg/kg DFMO, and 50 mg/kg HYD was 4% on day 8, 24.5% on day 12, and 27.3% on day 8, respectively.

**Fig 3:**
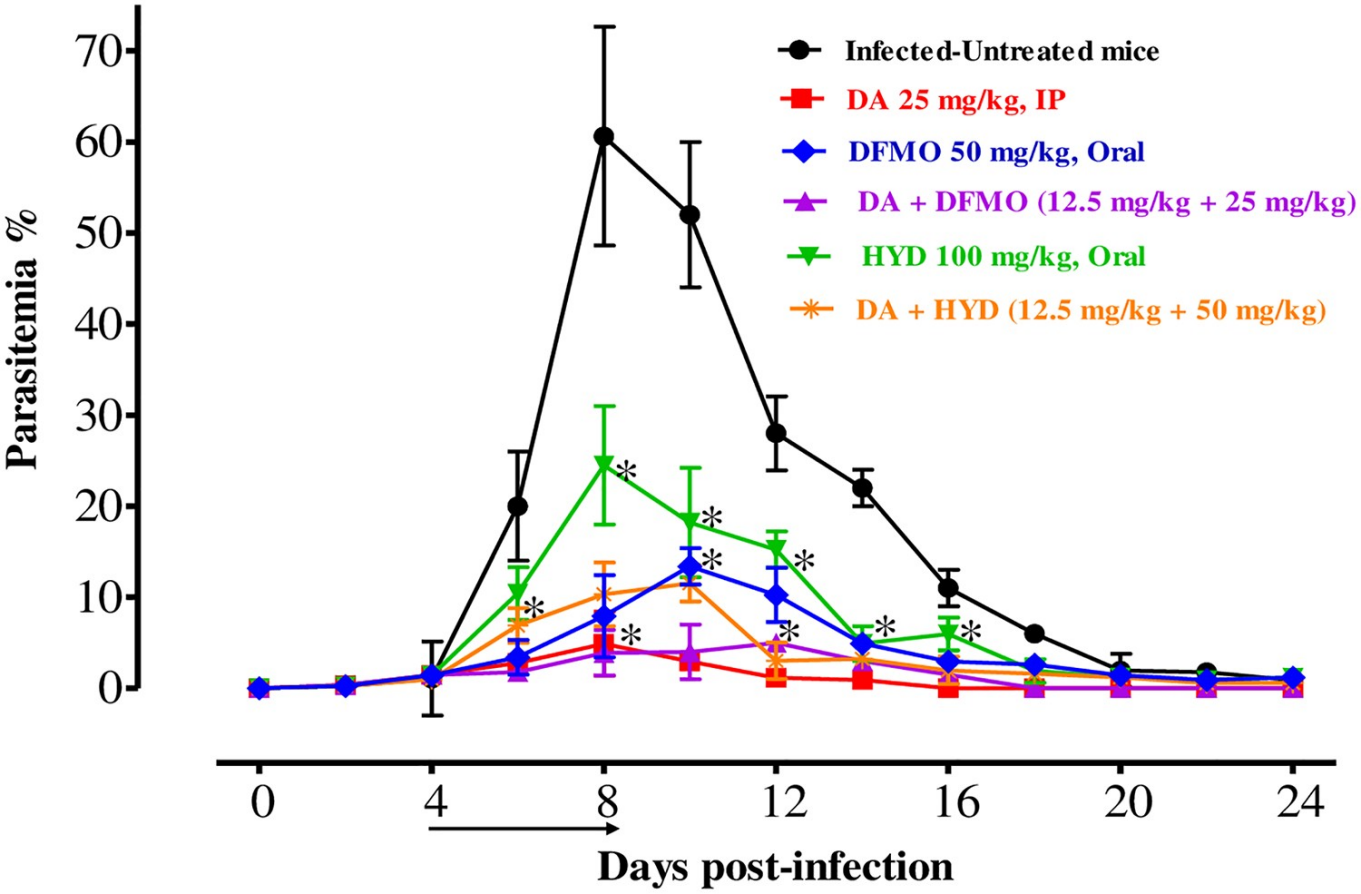
*In vivo* growth inhibition of HYD and DFMO against *B*. *microti*. Graph reveals the chemotherapeutic potential of HYD–DA and DFMO–DA when compared to the positive group. The asterisks (*) show significant variation (*P* < 0.05) between drug-treated and positive groups. The arrow shows 5 successive days of drug administration starting from day 4 to 8 p.i.

In the second assay, we changed the route of administration from intraperitoneal to oral for HYD and DFMO. On the eighth day p.i., the growth of *B*. *microti* in the DDW group was significantly enhanced and attained its highest parasitemia at 60.62%. The level of parasitemia in all treated groups was reduced at a statistically significant lower parasitemia percentage than that of the control group (ANOVA: *F*_(2.526, 11.11)_ = 5.264, *P* < 0.001 for monotherapy-treated groups; ANOVA: *F*_(3.458, 14.20)_ = 6.315, *P* < 0.0001 for combination-treated groups) from days 8 to 20 p.i. The level of peak parasitemia in the monochemotherapy-treated mice attained 3.9% on day 8, 13.2% on day 10, and 24.2% on day 8 in 25 mg/kg DA, 50 mg/kg DFMO, and 100 mg/kg HYD, respectively (Fig 3). According to microscopic examinations, parasitemia did not detect in groups treated with 25 mg/kg DA, 50 mg/kg DFMO, and 100 mg/kg HYD on days 16, 26, and 36 p.i., respectively. In addition to the DFMO inhibitory effect, it postponed the peak of parasitemia from day 8 to day 12, and thus seems to act more slowly than HYD and DA.

**Fig 4:**
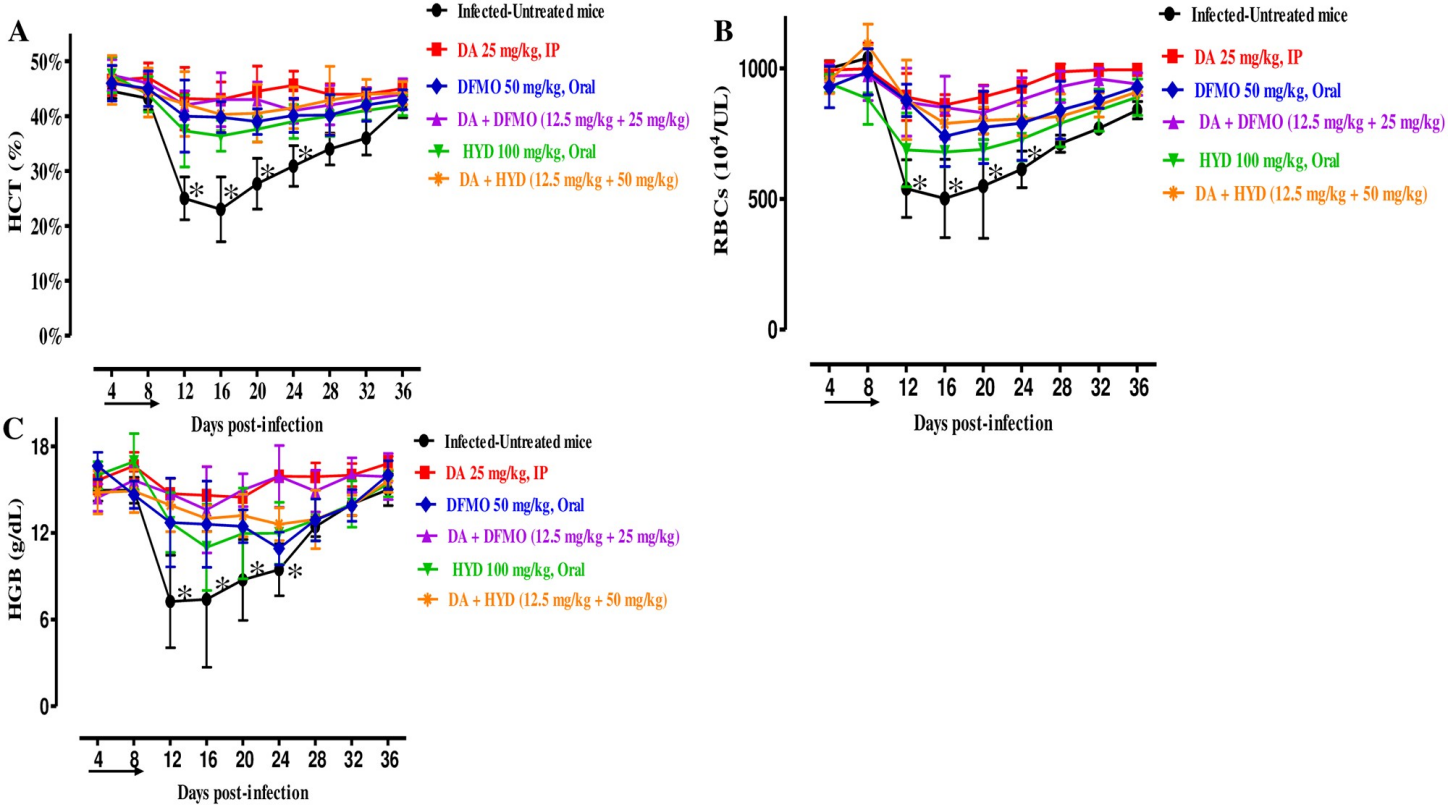
Hematology parameter changes in DFMO- and HYD-treated groups *in vivo*. Graphs showing the hematocrit (HCT) (A), red blood cells (RBCs) (B), and hemoglobin (HGB) (C) changes in treated mice compared to the infected-untreated mice. Asterisks (*) show significant variation (*P* < 0.05) between drug-treated and positive groups. The arrow shows 5 successive days of drug administration starting from day 4 to 8 p.i.

For the combination-treated groups, the levels of peak parasitemia exhibited 5% on day 12 in 25 mg/kg DFMO–12.5 mg/kg DA and 11.5% on day 10 in 50 mg/kg HYD–12.5 mg/kg DA (Fig 3). The first day on which parasitemia was not detected by microscopy was day 20 and 26 p.i. with DFMO–DA and HYD–DA, respectively. Furthermore, there are statistically significant differences in the HCT (ANOVA: *F*_(3.243, 14.32)_ = 5.697, *P* < 0.0001), RBC (ANOVA: *F*_(2.408, 13.6)_ = 5.876, *P* < 0.0001), and HGB concentration (ANOVA: *F*_(2.950, 16.61)_ = 5.681, *P* < 0.0001) detected between the drug-treated groups and DDW group on 8 and 12 days (Fig 4A–4C).

## Discussion

The current study revealed that HYD and DFMO inhibited the *in vitro* multiplication of piroplasm parasites. However, HYD’s highest efficacy was against *T*. *equi*. The efficacy of HYD and DFMO against tested parasites was comparable with previous reports that showed that HYD and DFMO have a potent efficacy on the intracellular protozoa elimination including *Leishmania*, *Toxoplasma*, and *Trypanosoma* parasites [14, 17, 29, 30, 31], thereby further establishing that HYD and DFMO were effective against many protozoan parasites. The IC_50_ values obtained from HYD and DFMO were lower than that showed by N-acetyl-L-cysteine [32], Allicin [33], thymoquinone against equine piroplasms parasites [34], norfloxacin, ofloxacin [35], *trans*-chalcone and chalcone hydrate against *B*. *divergens* [3], clodinafop-propargyl [36] and chalcone hydrate against bovine *Babesia* [3], fusidic acid against *B*. *bovis* [37] and ivermectin against *B*. *bigemina* [4]. While their IC_50_ values were higher than that of ellagic acid [7], nitidine chloride and camptothecin [5] and 17-DMAG [6].

Nowadays, combination chemotherapies are being reported to alleviate serious diseases, including pulmonary tuberculosis, malignancy, immune deficiency syndrome, and some protozoal diseases to promote higher therapeutic efficacy [6, 38]. Therefore, the present study examined the *in vitro* combination efficacy of HYD or DFMO with three other drugs—ATV, DA, and CLF. These results indicated that the effects of HYD or DFMO, when coupled with CLF, ATV, or, DA were additive or synergistic against the five tested parasites. Interestingly, previous reports documented the ability of HYD and DFMO to combine with other antiparasitic agents. For instance, Singh et al. [10] documented that the combination therapy of HYD with the heme biosynthesis inhibitor sampangine, several Erg11 inhibitors, and the antifungal azoles showed synergistic effect and these combinations could be a new approach for fungal infections treatment. Keithly et al. [30] have been reported that DFMO could suppress the parasitemia in *L*. *donovani*–or *L*. *braziliensis*–infected mice and the combined treatment of DFMO with other antileishmanial agents showed synergistic, additive, or no antiparasitic effects. Furthermore, combination treatment of eflornithine with nifurtimox has been used for the elimination and relief from second-stage African trypanosomiasis (sleeping sickness) caused by *T*. *brucei gambiense* [31]. However, it worth noting that additional researches should be conducted soon to emphasize how HYD and DFMO can act against piroplasm parasites to open the way to understanding the efficacy of interactions with existing babesicidal drugs.

The CCK test was used to examine the cytotoxicity of HYD and DFMO on animal and human cell lines, the main hosts of piroplasm parasites. Our results revealed that both compounds showed no inhibition on NIH/3T3 or HFF cell viability, whereas HYD only affected the MDBK cell viability at a high concentration. Additionally, pre cultivation of bovine and equine RBCs with HYD or DFMO *in vitro* detected that treated RBCs were unaffected with both drugs either morphologically or functionally. Previous reports showed that intravenous injection of DFMO at 200 mg/kg two times per day for two weeks had been documented as initial therapy for the sleeping disorder without signs of toxicity [39]. Melo and Beiral [40] reported that HYD strongly interfered with *L*. *amazonensis*, *T*. *gondii*, and *T*. *cruzi* multiplication, resulting in an irreversible morphological effect without affecting the host cells.

The *in vitro* inhibitory effects of HYD and DFMO motivated us for the assessment of their chemotherapeutic potential on *B*. *microti* infection in mice, and we found that they were indeed effective in this context as well. Significant reductions in HCT, RBCs, and HGB were detected in the positive control group. Interestingly, our findings are consistent with a recent study that showed that 17-DMAG led to lower hematological profile values similar to those of our [6]. Nevertheless, HYD and DFMO, like DA, prohibited anemia development in mice, although temporal reductions were observed in HCT, RBCs, and HGB. Furthermore, neither the HYD nor the DFMO treatment had any apparent toxic symptoms or promoted anemia in uninfected mice. This was consistent with previous reports documented that daily administration of 200–300 mg of HYD and DFMO up to 30 months have been successfully used in treating drug-resistant anemia, trypanosomiasis, and *Leishmania* in humans [41, 42], suggesting the safety of HYD and DFMO for use in clinical trials.

Although DA is the most effective babesicidal drug used in the veterinary field, it was unable to clear all parasites from the host animals. As a result, the disease can recur in treated animals. Moreover, restlessness, tissue injury at the site of injection, and abdominal pain have been observed in animals after the treatment of DA [43]. Therefore, a good combinatorial babesicidal drug is urgently needed. The *in vivo* experiment revealed that *B*. *microti* was cleared from HYD- and DFMO-treated mice. The oral administration of HYD and DFMO exhibited chemotherapeutic effect higher than the 34%, 31%, 49%, 58.3%, 37%, and 49% shown by enoxacin, norfloxacin, and ofloxacin [35], allicin [33], thymoquinone [34], and ellagic acid [7], respectively. Whereas, oral administration of HYD and DFMO exhibited chemotherapeutic effect lower than the 89% and 91% shown by 17-DMAG [6] and nitidine chloride [5], respectively but similar to that shown by *trans*-chalcone [3]. Interestingly, the combination treatment at a half-dose of HYD or DFMO with DA exhibited a strong chemotherapeutic efficacy similar to the full dose of a single drug. Since DFMO and HYD are typically used as part of multidrug therapies to treat several parasites [10, 31], this result reinforces that they are good combinatorial drugs. However, the synergetic and additive effects between the most common antipiroplasmic drugs (azithromycin, clindamycin, and imidocarb) and HYD and DFMO were not evaluated against the growth of *B*. *microti* in mice. Therefore, future studies are required to assess these combined effects either *in vitro* or *in vivo*.

Moreover, HYD and DFMO have been documented to be useful in chemotherapy for anemia, and sleeping sickness in humans [16, 22] and the present study showed their efficacy against mice infected with *B*. *microti*, and thus, they might be used as an alternative chemotherapy for humans infected with *B*. *microti* after some clinical studies. However, PCR assay should be used alongside optical microscopy in future similar works to detect parasites DNA from *in vitro* cultures as well as from peripheral blood of animal models *in vivo* to ensure a total clearance of parasites. Moreover, additional research is urgently needed to assess the efficacy of HYD or DFMO when combined with ATV or CLF *in vivo* as well as to elucidate their mechanism of action against piroplasm parasites.

## Conclusions

To our knowledge, this is the first antipiroplasmic evaluation of HYD and DFMO against piroplasm parasites. HYD and DFMO exhibited an *in vitro* growth inhibitory effect against five piroplasm species as well as chemotherapeutic efficacy toward *B*. *microti in vivo*. Furthermore, the combination treatment of HYD and DFMO with DA, ATV, and CLF demonstrated synergistic and additive efficacy against all tested parasites. These results are implying that they possess potential value for treating clinic diseases in animals and humans either alone or in combination with other drugs.

## Supporting information

S1 Fig Growth of *B*. *bovis* with HYD- or DFMO-treated bovine RBCs. (TIF)

S2 Fig Growth of *T*. *equi* with HYD- or DFMO-treated horse RBCs. (TIF)

S1 Table Concentrations of HYD and DFMO combined with DA, ATV, and CLF against *Babesia* and *Theileria* parasites *in vitro*. (DOCX)

S2 Table The effect of HYD and DFMO combined with DA, ATV, and CLF against *Babesia* and *Theileria* parasites *in vitro*. (DOCX)

S3 Table IC_50_ and selective index values of DA, ATV, and CLF. (DOCX)

## Abbreviations

ATV: Atovaquone
CCK-8: Cell Counting Kit-8
CI: Combination index
CLF: clofazimine
DA: Diminazene aceturate
DDW: Double distilled water
DFMO: Eflornithine (α-difluoro-methyl ornithine)
DMEM: Dulbecco’s Modified Eagle’s Medium
DMSO: Dimethyl sulfoxide
EC_50_: Half maximum effective concentrations
EDTA: Ethylenediaminetetraacetic acid
HCT: Hematocrit
HFF: Human foreskin fibroblast
HGB: Hemoglobin
HYD: Hydroxyurea
IC_50_: Half maximal inhibitory concentration
iRBCs: infected red blood cells
M199: Medium 199
MDBK: Madin-Darby bovine kidney
MEM: Minimum Essential Medium Eagle
NIH/3T3: Mouse embryonic fibroblast
RBCs: Red blood cells
