# Supplementary figures and images for: Safety and efficacy of hydroxyurea and eflornithine against most blood parasites Babesia and Theileria
Source: PLoS One. 2020 Feb 13;15(2):e0228996. doi: 10.1371/journal.pone.0228996 (PMC7018007; doi:10.1371/journal.pone.0228996)

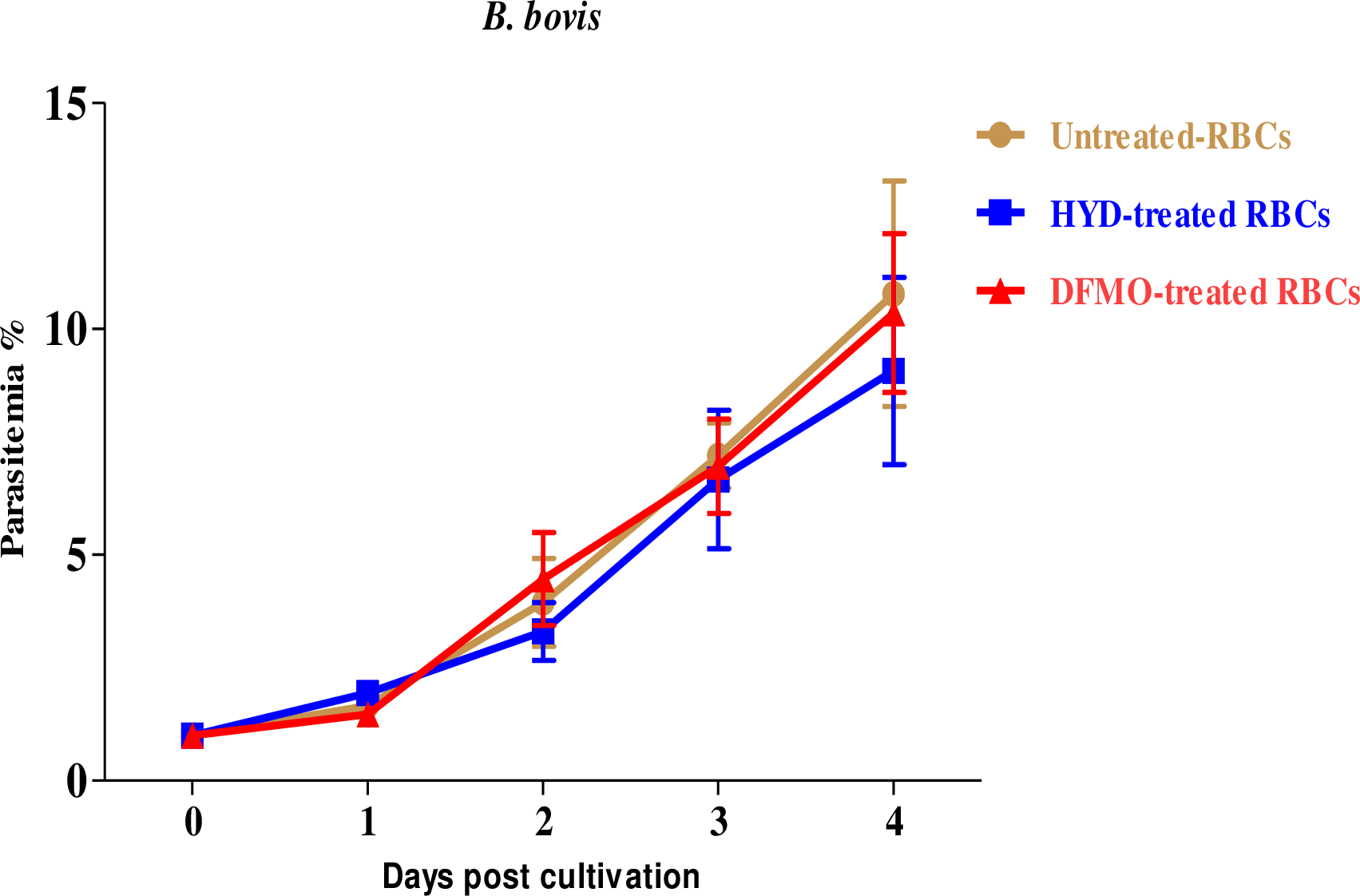

Supplement: S1 Fig — (TIF) [file pone.0228996.s001.tif]

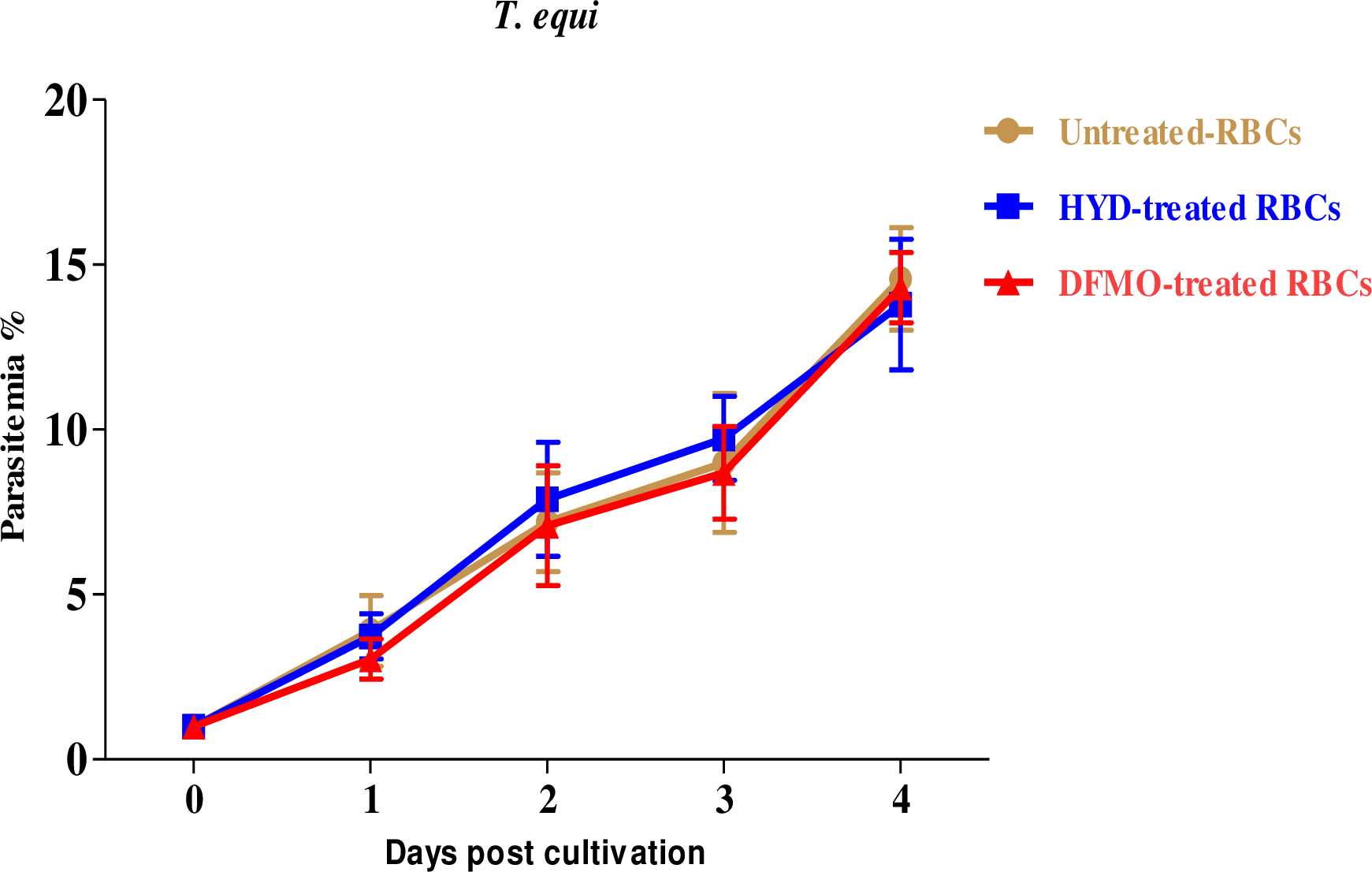

Supplement: S2 Fig — (TIF) [file pone.0228996.s002.tif]
