# Supplementary material for: Safety and efficacy of hydroxyurea and eflornithine against most blood parasites Babesia and Theileria
Source: PLoS One. 2020 Feb 13;15(2):e0228996. doi: 10.1371/journal.pone.0228996 (PMC7018007; doi:10.1371/journal.pone.0228996)
Supplement: S1 Table — (DOCX) [file pone.0228996.s003.docx]

**S1 Table. Concentrations of HYD or DFMO combined with DA, ATV, or CLF against *Babesia* and *Theileria* parasites *in vitro***

| **Parasite** | **Concentration** | **HYD** | **DFMO** | **DA** | **ATV** | **CLF** |
| --- | --- | --- | --- | --- | --- | --- |
| ***B. bovis*** | **N_1_** | 21.3 | 24.5 | 0.0875 | 0.00975 | 2.06 |
|  | **N_2_** | 42.6 | 49 | 0.175 | 0.0195 | 4.12 |
|  | **N_3_** | 85.2 | 98 | 0.35 | 0.039 | 8.24 |
|  | **N_4_** | 170.4 | 196 | 0.7 | 0.078 | 16.48 |
|  | **N_5_** | 340.8 | 392 | 1.4 | 0.156 | 32.96 |
|  |  |  |  |  |  |  |
| ***B. bigemina*** | **N_1_** | 17.175 | 19.75 | 0.17 | 0.17525 | 1.4325 |
|  | **N_2_** | 34.35 | 39.5 | 0.34 | 0.3505 | 2.865 |
|  | **N_3_** | 68.7 | 79 | 0.68 | 0.701 | 5.73 |
|  | **N_4_** | 137.4 | 158 | 1.36 | 1.402 | 11.46 |
|  | **N_5_** | 274.8 | 316 | 2.72 | 2.804 | 22.92 |
|  |  |  |  |  |  |  |
| ***B. divergens*** | **N_1_** | 14.325 | 11.725 | 0.1075 | 0.0095 | 3.4625 |
|  | **N_2_** | 28.65 | 23.45 | 0.215 | 0.019 | 6.925 |
|  | **N_3_** | 57.3 | 46.9 | 0.43 | 0.038 | 13.85 |
|  | **N_4_** | 114.6 | 93.8 | 0.86 | 0.076 | 27.7 |
|  | **N_5_** | 229.2 | 187.6 | 1.72 | 0.152 | 55.4 |
|  |  |  |  |  |  |  |
| ***B. caballi*** | **N_1_** | 12.375 | 17.75 | 0.0055 | 0.0255 | 1.9875 |
|  | **N_2_** | 24.75 | 35.5 | 0.011 | 0.051 | 3.975 |
|  | **N_3_** | 49.5 | 71 | 0.022 | 0.102 | 7.95 |
|  | **N_4_** | 99 | 142 | 0.044 | 0.204 | 15.9 |
|  | **N_5_** | 198 | 284 | 0.088 | 0.408 | 31.8 |
|  |  |  |  |  |  |  |
| ***T. equi*** | **N_1_** | 4.9 | 24.25 | 0.775 | 0.02375 | 0.72 |
|  | **N_2_** | 9.8 | 48.5 | 0.355 | 0.0475 | 1.44 |
|  | **N_3_** | 19.6 | 97 | 0.71 | 0.095 | 2.88 |
|  | **N_4_** | 39.2 | 194 | 1.42 | 0.19 | 5.76 |
|  | **N_5_** | 78.4 | 388 | 2.84 | 0.38 | 11.52 |

N_1_–N_5_ refer to the concentrations (**µM**) of HYD or DFMO combined with DA, ATV, or CLF. Combined concentrations were based on the calculated IC_50_ values obtained from the *in vitro* fluorescence-based assay

*Abbreviations: HYD,* hydroxyurea; *DFMO,* eflornithine; *DA,* diminazene aceturate; *ATV,* atovaquone; *CLF,* clofazimine
