# Supplementary material for: Safety and efficacy of hydroxyurea and eflornithine against most blood parasites Babesia and Theileria
Source: PLoS One. 2020 Feb 13;15(2):e0228996. doi: 10.1371/journal.pone.0228996 (PMC7018007; doi:10.1371/journal.pone.0228996)
Supplement: S2 Table — (DOCX) [file pone.0228996.s004.docx]

**S2 Table. The effects of HYD or DFMO with DA, ATV, or CLF against *Babesia* and *Theileria* parasites *in vitro***

| **Degree of association ^c^** | **Weighted average CI value ^b^** | **CI values** | | | | **Drug combination ^a^** | **Parasite** |
| --- | --- | --- | --- | --- | --- | --- | --- |
|  |  | **IC_95_** | **IC_90_** | **IC_75_** | **IC_50_** |  |  |
| **Additive**  **Additive** | 1.1014  1.101 | 1.072  1.026 | 1.101  1.082 | 1.273  1.425 | 1.010  1.200 | **HYD+DA**  **DFMO+DA** | ***B. bovis*** |
| **Additive**  **Synergistic**  **Synergistic**  **Synergistic** | 1.0455  0.3873  0.6171  0.2771 | 1.235  0.511  0.970  0.261 | 0.962  0.322  0.277  0.129 | 0.945  0.321  0.280  0.290 | 0.739  0.219  0.900  0.760 | **HYD+ATV**  **DFMO+ATV**  **HYD+CLF**  **DFMO+CLF** |  |
| **Synergistic**  **Synergistic** | 0.8336  0.6712 | 0.921  0.961 | 0.876  0.671 | 0.912  0.291 | 0.236  0.273 | **HYD+DA**  **DFMO+DA** | ***B. bigemina*** |
| **Synergistic**  **Synergistic**  **Synergistic**  **Additive** | 0.3470  0.5180  0.5692  1.104 | 0.195  0.532  0.781  1.040 | 0.296  0.291  0.568  1.409 | 0.652  0.981  0.318  1.009 | 0.498  0.217  0.228  1.199 | **HYD+ATV**  **DFMO+ATV**  **HYD+CLF**  **DFMO+CLF** |  |
| **Synergistic**  **Additive** | 0.8502  1.2231 | 1.200  1.002 | 1.008  1.421 | 1.107  1.470 | 1.002  1.021 | **HYD+DA**  **DFMO+DA** | ***B. divergens*** |
| **Synergistic**  **Synergistic**  **Synergistic**  **Synergistic** | 0.7046  0.8730  0.4342  0.6158 | 0.772  0.953  0.785  0.800 | 1.080  0.901  0.557  0.153 | 1.007  1.081  0.857  0.813 | 0.704  0.053  0.957  0.873 | **HYD+ATV**  **DFMO+ATV**  **HYD+CLF**  **DFMO+CLF** |  |
| **Synergistic**  **Synergistic** | 0.7512  0.8520 | 0.698  1.072 | 0.667  1.370 | 0.896  1.052 | 0.927  0.082 | **HYD+DA**  **DFMO+DA** | ***B. caballi*** |
| **Synergistic**  **Synergistic**  **Additive**  **Additive** | 0.8164  0.7348  0.9126  1.0238 | 0.779  0.783  1.098  0.963 | 0.862  0.491  0.732  1.159 | 0.789  0.953  0.712  0.858 | 0.884  0.837  1.114  1.193 | **HYD+ATV**  **DFMO+ATV**  **HYD+CLF**  **DFMO+CLF** |  |
| **Synergistic**  **Synergistic** | 0.7710  0.7022 | 0.572  0.963 | 1.099  0.192 | 0.582  0.829 | 0.961  0.936 | **HYD+DA**  **DFMO+DA** | ***T. equi*** |
| **Synergistic**  **Synergistic**  **Synergistic**  **Synergistic** | 0.6750  0.6589  0.8150  0.5470 | 0.942  0.358  0.786  0.932 | 0.495  0.938  0.865  0.396 | 0.639  0.798  1.005  0.887 | 0.219  0.747  0.915  0.497 | **HYD+ATV**  **DFMO+ATV**  **HYD+CLF**  **DFMO+CLF** |  |

CI value, combination index value; IC_50_, 50% inhibition concentration. ^a^ Two-drug combination of HYD or DFMO with DA, ATV, or CLF at a concentration of approximately 0.25×IC_50_, 0.5×IC_50_, IC_50_, 2×IC_50_, and 4×IC_50_ (constant ratio). ^b^The higher inhibition is preferable, thus the weighted average CI value was calculated using the formula [(1×IC_50_) + (2×IC_75_) + (3×IC_90_) + (4×IC_95_)]/10. ^c^The degree of synergism was determined based on the following CI value: < 0.90 (synergistic), 0.90–1.10 (additive), and > 1.10 (antagonistic)

*Abbreviations: HYD,* hydroxyurea; *DFMO,* eflornithine; *DA,* diminazene aceturate; *ATV,* atovaquone; *CLF,* clofazimine; *CI,* combination index
