# Supplementary material for: Safety and efficacy of hydroxyurea and eflornithine against most blood parasites Babesia and Theileria
Source: PLoS One. 2020 Feb 13;15(2):e0228996. doi: 10.1371/journal.pone.0228996 (PMC7018007; doi:10.1371/journal.pone.0228996)
Supplement: S3 Table — (DOCX) [file pone.0228996.s005.docx]

**S3 Table. IC_50_ and selective index values of DA, ATV and CLF**

| **Drug** | ***Babesia* and *Theileria*** | **IC_50_ (µM)^a^** | **EC_50_ (µM)^b^** | | | **Selective index^c^** | | |
| --- | --- | --- | --- | --- | --- | --- | --- | --- |
|  |  |  | **MDBK** | **NIH/3T3** | **HFF** | **MDBK** | **NIH/3T3** | **HFF** |
| **DA** | *B. bovis* | **0.35 ± 0.06** | **˃100** | **˃100** | **˃100** | **˃ 285.7** | **˃ 285.7** | **˃ 285.7** |
|  | *B. bigemina* | **0.68 ± 0.09** | **˃100** | **˃100** | **˃100** | **˃ 147.1** | **˃ 147.1** | **˃ 147.1** |
|  | *B. divergens* | **0.43 ± 0.05** | **˃100** | **˃100** | **˃100** | **˃ 232.5** | **˃ 232.5** | **˃ 232.5** |
|  | *B. caballi* | **0.02 ± 0.0002** | **˃100** | **˃100** | **˃100** | **˃ 5000** | **˃ 5000** | **˃ 5000** |
|  | *T. equi* | **0.71 ± 0.05** | **˃100** | **˃100** | **˃100** | **˃ 140.8** | **˃ 140.8** | **˃ 140.8** |
| **ATV** | *B. bovis* | **0.039 ± 0.002** | **˃100** | **˃100** | **˃100** | **˃ 2564.1** | **˃ 2564.1** | **˃ 2564.1** |
|  | *B. bigemina* | **0.701 ± 0.04** | **˃100** | **˃100** | **˃100** | **˃ 142.7** | **˃ 142.7** | **˃ 142.7** |
|  | *B. divergens* | **0.038 ± 0.002** | **˃100** | **˃100** | **˃100** | **˃ 2631.6** | **˃ 2631.6** | **˃ 2631.6** |
|  | *B. caballi* | **0.102 ± 0.014** | **˃100** | **˃100** | **˃100** | **˃ 980.4** | **˃ 980.4** | **˃ 980.4** |
|  | *T. equi* | **0.095 ± 0.065** | **˃100** | **˃100** | **˃100** | **˃ 1052.6** | **˃ 1052.6** | **˃ 1052.6** |
| **CLF** | *B. bovis* | **8.24 ± 1.7** | **34.72 ± 3.4** | **˃100** | **˃100** | **4.2** | **˃ 12.1** | **˃ 12.1** |
|  | *B. bigemina* | **5.73 ± 1.9** | **34.72 ± 3.4** | **˃100** | **˃100** | **6.1** | **˃ 17.5** | **˃ 17.5** |
|  | *B. divergens* | **13.85 ± 4.3** | **34.72 ± 3.4** | **˃100** | **˃100** | **2.5** | **˃ 7.2** | **˃ 7.2** |
|  | *B. caballi* | **7.95 ± 1.8** | **34.72 ± 3.4** | **˃100** | **˃100** | **4.4** | **˃ 12.6** | **˃ 12.6** |
|  | *T. equi* | **2.88 ± 0.9** | **34.72 ± 3.4** | **˃100** | **˃100** | **12.1** | **˃ 34.7** | **˃ 34.7** |

^a^ IC_50_ values of DA, ATV and CLF on all tested parasites *in vitro*. ^b^ EC_50_ values of DA, ATV and CLF on the tested cell lines. The dose-response curve using nonlinear regression (curve fit analysis) was used to detect all these values. The values obtained from the means of triplicate experiments. ^c^ Selective index calculated as the ratio of the EC_50_ of cell lines to the IC_50_ of each parasite.
